# Intrinsic Radiosensitivity Is Not the Determining Factor in Treatment Response Differences between HPV Negative and HPV Positive Head and Neck Cancers

**DOI:** 10.3390/cells9081788

**Published:** 2020-07-27

**Authors:** Paul Reid, Alexander H. Staudacher, Loredana G. Marcu, Ian Olver, Leyla Moghaddasi, Michael P. Brown, Yanrui Li, Eva Bezak

**Affiliations:** 1 School of Health Sciences, University of South Australia, Adelaide, SA 5001, Australia; loredana@marcunet.com (L.G.M.); Eva.Bezak@unisa.edu.au (E.B.); 2 Cancer Research Institute, University of South Australia, Adelaide, SA 5001, Australia; judy.li@unisa.edu.au; 3 Translational Oncology Laboratory, Centre for Cancer Biology, SA Pathology and University of South Australia, Adelaide, SA 5000, Australia; alex.staudacher@health.sa.gov.au (A.H.S.); MichaelP.Brown@sa.gov.au (M.P.B.); 4 School of Psychology, University of Adelaide, Adelaide, SA 5000, Australia; ian.olver@adelaide.edu.au; 5 Faculty of Science, University of Oradea, 410087 Oradea, Romania; 6 Department of Physics, University of Adelaide, Adelaide, SA 5005, Australia; Leyla.Moghaddasi@genesiscare.com; 7 Genesis Care, Adelaide Radiotherapy Centre, Adelaide, SA 5000, Australia; 8 School of Medicine, University of Adelaide, Adelaide, SA 5000, Australia; 9 Cancer Clinical Trials Unit, Royal Adelaide Hospital, Adelaide, SA 5000, Australia

**Keywords:** head and neck cancer, HNSCCC, HPV, radiosensitivity, clonogenic assay, fractionated radiation therapy

## Abstract

Head and neck squamous cell carcinomas (HNSCC) resulting from human papillomavirus (HPV) are increasing in incidence but demonstrate significantly better treatment response than HNSCC from other causes such as tobacco and alcohol. This study sought to identify differences in HNSCC, intrinsic to HPV status, in their response to radiation dose. Previously unexamined changes in radio-responsiveness following fractionated X-ray irradiation were compared between HPV positive and negative statuses of HNSCC. Six HNSCC cell lines, 3 of each HPV status, were investigated for radiosensitivity by clonogenic assay and modelled by response as a function of dose. Generational cultures of each cell line were developed to follow changes in radiosensitivity after repeated irradiations simulating fractionated radiation therapy. As a group, the HPV positive cell lines were more radiosensitive, but with changes following repeated fractions of dose, and modelling of response as a function of dose, both statuses displayed large radiobiological heterogeneity. These findings challenge current radiobiological assumptions of head and neck cancers as early responding tissue to radiation and may go some way in explaining difficulties reaching consensus in stratification of treatment by HPV status. Consequently, results from this study do not support stratifying radiation therapy by HPV status.

## 1. Introduction

Head and neck cancers are heterogeneous, occurring from the oral and nasal cavities to the larynx and pharyngeal areas where about 95% are squamous cell carcinomas (HNSCC) of the mucosal epithelium [1]. Importantly, causes of head and neck cancers fall into 2 main etiological groups, being HNSCC resulting from human papillomavirus (HPV) infection, and those resulting from other causes such as tobacco and alcohol [2]. Oropharyngeal squamous cell carcinoma (OPSCC) represents a growing subset of HNSCC due to the increasing incidence of HPV driven tumors, and the preference shown by HPV for this anatomical area [3,4]. Diagnosis of HPV positive HNSCC is typically in a younger demographic and its rise in incidence has now overtaken HPV associated cervical cancers, stressing the growing health concern and emphasis on research efforts to understand the best approaches to the control of the 2 etiological groups [5]. Treatment frequently involves a multimodality approach where around 75% of patients will undergo some form of radiation therapy [6]. Importantly, the HPV positive status demonstrates a consistent and significantly better response to treatment, establishing it as a positive prognostic maker [7,8,9]. The increasing frequency of HPV positive HNSCC in younger patients also makes it crucial to facilitate more selective treatment for these patients. To date, efforts to greater individualize treatments have been aimed chiefly at dose de-escalation in the HPV positive group, to achieve minimal normal tissue effects [10,11,12]. Treatment protocols for HNSCC however, continue to be largely the same irrespective of HPV status [3,11]. Moves to use HPV status as a marker for treatment stratification require a more predictive understanding of response to radiation dose in terms of HPV status.

The use of clonogenic assays to determine radiosensitivity remains a gold standard in radiation biology. It determines cellular radiation sensitivity by surviving fractions (SF) of clonogens in cell cultures as a function of radiation dose delivered [13,14]. This response to radiation is traditionally described by the linear quadratic model (LQM):

(1) $$SF=exp^{-\left(\propto D+\beta D^{2}\right)}$$

where α is the coefficient of cell killing at low doses resulting from predominantly single radiation hits, and β is the coefficient of cell killing from multiple hits at higher doses (>2 Gy) [15]. The LQM and α, β parameters are also used in calculations of tumor control probability (TCP) and normal tissue complication probability (NTCP). Clinically, tumor irradiation is performed in a series of irradiations or fractions, delivering smaller radiation doses (typically 2 Gy per fraction) each time, until a total prescribed radiation dose is reached. Radiobiological parameters α, β are also used when comparing various dose-per-fraction irradiation schedules to determine the resulting biologically effective dose (BED) for a tissue [16]; i.e., the radiation dose that will result in the same biological endpoint for differently fractionated irradiations. These parameters, derived from the LQM as a ratio (α/β), describe the response of the tissue to radiation as early or late responding. Late responding tissue shows less cell death at low doses (<2 Gy) but cell killing accelerates more quickly than in early responding tissue for higher doses. Late responding tissues have a low α/β (<6 Gy) indicating greater change in cell killing with alteration in the fractionation of a total radiation dose [17,18]. This radio-responsiveness results in greater fractional sensitivity. Tissues with high α/β (>6 Gy) display less change in cell killing with alteration in the fractionation of the total radiation dose and the contrast in responsiveness between the 2 tissue types means fractionation may be used selectively to enhance tumor cell killing while maintaining normal tissue sparing [18]. Biological endpoints, as a function of radiation dose, can be compared between fractionation schedules, and for different tissues, by the BED [19]:

(2) $$BED=nd\left[1+\frac{d}{\alpha /\beta }\right]$$

where *n* is the number of fractions and *d* is dose per fraction.

Some radiobiological studies of HNSCC report HPV positive cancers to be more radiosensitive than HPV negative cancers which is consistent with clinical observations of better responses to radiotherapy [20,21,22]. However, head and neck cancers are also broadly heterogeneous in measures of radiosensitivity by LQM parameters, reflective of differences in anatomy, histology and etiology [17,23]. Additionally, potential changes in their radio-responsiveness during fractionated irradiation have not been measured and quantified.

As such, the aim of this study was to go beyond the single measure of radiosensitivity and compare changes in intrinsic HNSCC cellular radio-responsiveness according to HPV status, following repeated fractional radiation doses, thus simulating the clinical scenario in radiation therapy. To facilitate this, “generational cell cultures” were developed from HNSCC cell lines in the current work to investigate post-irradiation changes in radio-responsiveness.

This is the first-time HNSCC cell lines were investigated to compare treatment response following fractionated irradiation, enabling comparison of the radiobiology intrinsic to HPV status. Evaluations of response to radiation dose are made by the SF of cell population and by α/β ratios for each cell line. Data on changing radio-responsiveness following multiple dose fractions presents a further dimension to the radiobiology of these cell lines. This study illustrates the complexity of using HPV status for predicting response to irradiation.

## 2. Materials and Methods

### 2.1. Cell Culture

Three cell lines representing each HPV status were selected. UM-SCC-47, UPCI-SCC-154, and UPCI-SCC-090 are HPV positive. UM-SCC-17a, UM-SCC-22a and UM-SCC-1 are HPV negative (Table 1). UM-SCC-22a, UM-SCC-47, UM-SCC-17a and UM-SCC-1 were sourced through Merck Millipore (Darmstadt, Germany). UPCI-SCC-154 (ATCC^®^ CRL-3241TM) and UPCI-SCC-090 (ATCC^®^ CRL-3239TM) were sourced from ATCC (Manassas, VA, USA). To reflect characteristic mutational status of the HPV groups, the chosen HPV negative cell lines have TP53 mutations and HPV positive retain wild type expression. Cell lines were grown in Dulbecco’s Modified Eagle’s Medium with 4500 mg/L glucose (Sigma-Aldrich^®^ Darmstadt, Germany) 10% fetal bovine serum (FBS), 10 mM HEPES, 100 U/mL penicillin and 0.1 mg/mL streptomycin (Sigma-Aldrich^®^ Darmstadt, Germany). Cell cultures were grown in T25 flasks (Greiner Bio-One, Frickenhausen, Germany) and incubated at 37 °C in humidified atmosphere with 5% CO_2_.

### 2.2. Irradiation Setup

Cells were irradiated as a monolayer in T25 flasks using an RS2000 X-ray cabinet irradiator (Rad Source, Buford GA) using a 160 kV, 25 mA X-ray beam with 9.1 mm Al half value layer [27]. The beam was calibrated by AAPM TG61 protocol [28]. Flasks were filled with media to achieve electronic equilibrium at the monolayer, and full radiation scatter was facilitated by encasement of flasks in Paraffin in addition to mounting on 7 cm of solid water (RW3; PTW, Freiburg DE; ρ = 1.0459 g/cm^3^). For clonogenic assays, one flask of each cell culture was irradiated to an absorbed radiation dose of 0.5, 1.0, 2.0, 4.0 and 6.0 Gy, respectively. A 6th flask was sham irradiated as control. An additional flask was irradiated to 4 Gy for growing the next generation of cells to simulate fractionated radiation therapy (see below).

### 2.3. Clonogenic Assay

Following irradiation, cells were trypsinized and counted prior to plating in 6 well plates (Falcon, Corning, NY, USA) previously coated with extracellular matrix (Matrigel, Corning, NY, USA). One plate (6 repeat assays) for each dose point was used, with 1.5 mL of a 1:1 mix of culture media and conditioned media (filtered media from 1st generation cell cultures) per well, then incubated 10 to 14 days. Resulting colonies were fixed with 1:5 acetic acid/methanol solution before staining with 0.5% crystal violet. Clonogens were identified microscopically by colonies >50 cells and counted using ImageJ software v.1.51j [29] (U.S. National Institutes of Health, MD USA, https://imagej.nih.gov/ij/).

### 2.4. Generational Development

**Figure 1:**
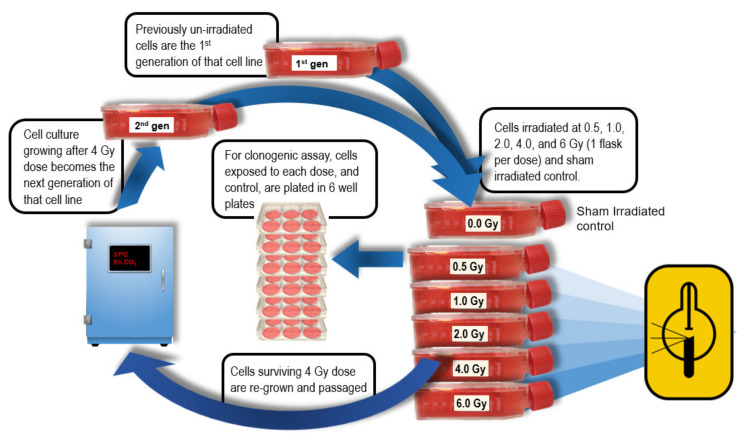
Generational development of cell line cultures following 4 Gy fractions. Cells from the T25 flask that received 4 Gy were re-cultured to become the next generation of that cell line then re-assayed.

To measure radiosensitivity of cell lines after repeated fractions, generational cultures of each line were developed as follows: Cells surviving 4 Gy irradiation (and not used in clonogenic assay) were re-cultured to grow the next (i.e., post-irradiation) generation of that cell line for subsequent irradiation and clonogenic assay (Figure 1). Four generations of each line were developed in this manner, except for UPCI-SCC-090 where only 2 generations were able to be grown. The 4 Gy radiation dose was chosen as the fractional dose over the usual clinical 2 Gy, to accelerate BED for the 4 generations irradiated, and to manifest differences in radiobiological response during fractionated irradiation.

### 2.5. Statistical Analysis and Modelling

Data from colony counting was analyzed in Microsoft Excel and Prism^®^ v8.2.1 (GraphPad Software Inc., La Jolla, CA, USA). SF as a function of absorbed dose, was determined from colony numbers (6 repeats per irradiation dose) by calculating the plating efficiency (PE) of controls and normalizing colony numbers as a function of dose to PE.

(3) $$PE=\left(\frac{number\;of\;colonies}{number\;of\;cells\;plated}\right)\times 100$$

(4) $$SF=\left(\frac{number\;of\;colonies}{PE}\right)\times 100$$

Survival curves were plotted, and α/β ratios fitted from SF using the LQM in MATLAB vR2019a.

## 3. Results

### 3.1. Comparison of Survival Fractions between HPV Groups

**Figure 2:**
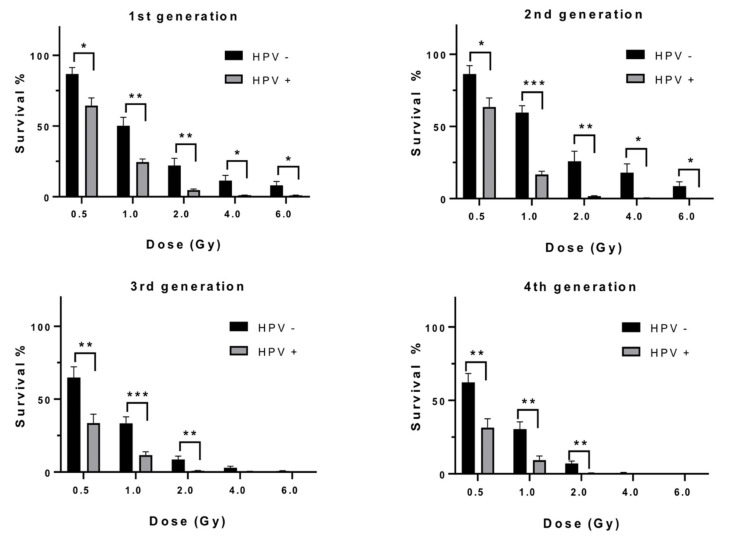
Surviving fractions of HPV negative versus HPV positive HNSCC cells, as a function of dose for each generational group. Error bars shown as standard error of the mean (SEM), 3 lines per HPV status. Significance, *p* values, * ≤ 0.05, ** ≤ 0.01, *** ≤ 0.001.

Comparing SF for each dose between HPV positive and negative cell line groups demonstrated significantly greater survival at each dose for the HPV negative cell line group in the first 2 generations and in the absorbed dose range of 0.5–2.0 Gy for the 3rd and 4th generations. The most significant differences in radiosensitivity among cell line groups defined by HPV status was at 1.0 and 2.0 Gy absorbed dose. Survival of HPV negative cells for 1.0 Gy was approximately 2-fold greater than HPV positive cells and 4-fold at 2 Gy absorbed dose. There was little overall change in SF differences according to HPV status over the 4 generations (Figure 2).

**Figure 3:**
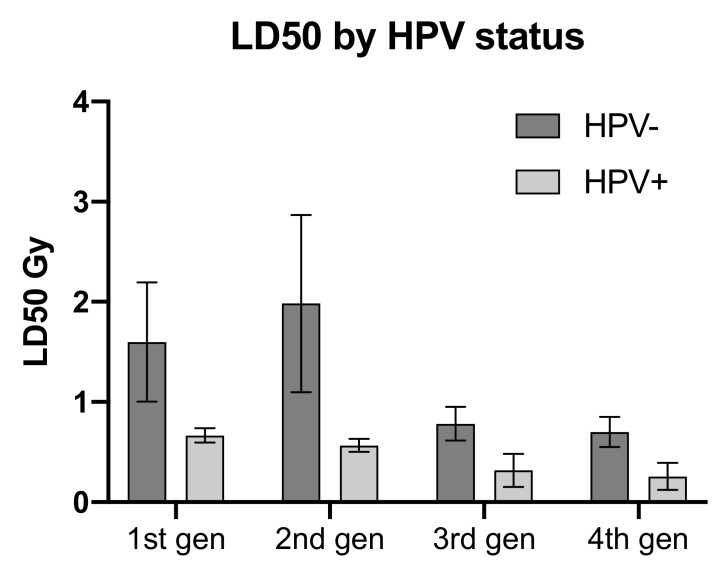
Comparison of dose resulting in 50% lethality (LD500 by HPV status. Error bars shown as SEM, 3 lines per HPV status.

Comparison of the dose to 50% lethality (LD50) by HPV status, also demonstrated greater radiosensitivity in the HPV positive cell lines. Although not statistically significant, the difference in sensitivity was evident for all generations of cell lines shown in Figure 3.

### 3.2. Changes in Radiosensitivity Following Repeated 4 Gy Fractions

**Figure 4:**
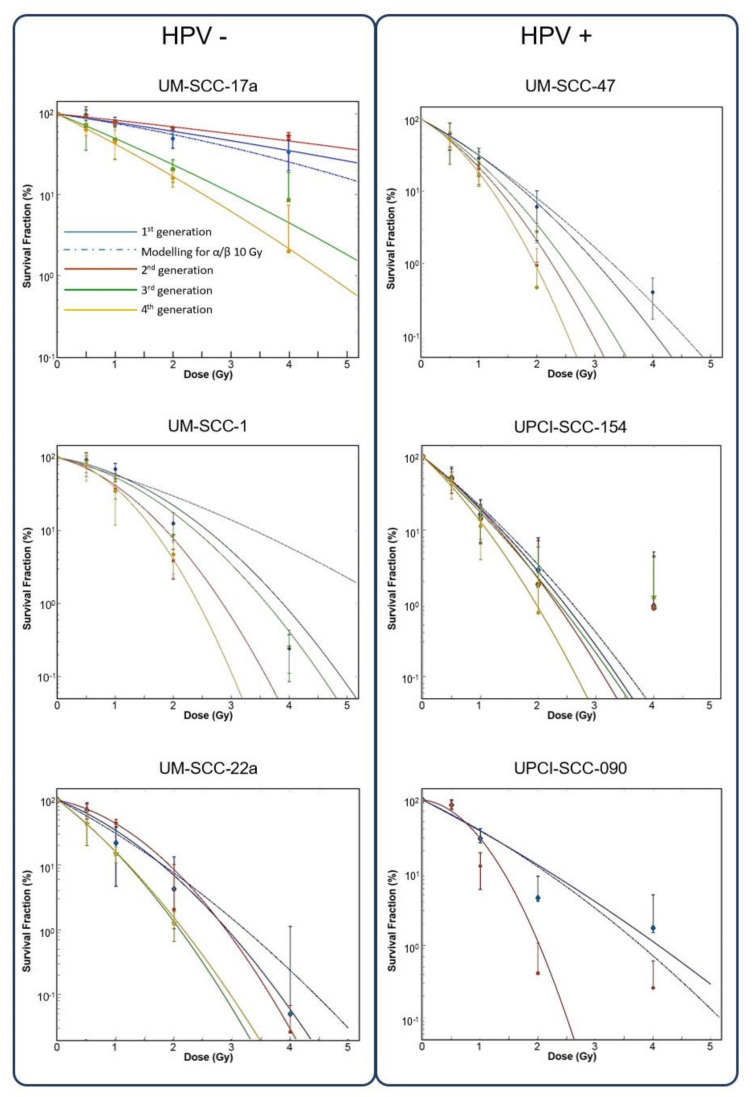
Survival curves for up to 4 generations of each cell line by HPV status. Dotted blue line shows 1st generation data modelling to HNSCC standard α/β ratio of 10 Gy. Clonogen SF shown on Y axis (log 10) as a function of absorbed dose (x axis).

Only the HPV negative HNSCC cells as a group, displayed an increased SF for dose where the 2nd generation increased SF over the 1st generation by 18.5%, 16.5% and 58.5% for 1.0, 2.0 and 4.0 Gy absorbed doses respectively. Both HPV groups then demonstrated diminishing SF after the second generation (Figure 3). Cell lines individually however, exhibited a large degree of heterogeneity in radiosensitivity and changes in responsiveness with repeated 4.0 Gy irradiation fraction. Overall, the 6 cell lines displayed a trend to increasing radiosensitivity with repeated fractions, but this varied greatly between the cell lines of both HPV groups (Figure 4). Among the HPV negative lines, UM-SCC-17a proved the least radiosensitive with surviving colonies growing after 6 Gy in all 4 generations. The 2nd generation showed decreased sensitivity with a rise in SFs of 24, 17 and 20% for 1.0, 2.0, and 4.0 Gy absorbed doses respectively, where increases at 2.0 and 4.0 Gy were significant. UM-SCC-22a also displayed increasing SF in the 2nd generation of 5 and 22% for absorbed doses of 0.5 and 1.0 Gy, respectively. SFs in consecutive generations of UM-SCC-1 showed increasing sensitivity with repeated fractions. For significance of change in SF in consecutive generations please see Appendix A. The HPV positive lines did not at any stage exhibit an increase in SF in a subsequent generation for dose. There was, however, large variation in the changing sensitivity between lines. UPCI-SCC-154 and UM-SCC-47 showed mostly non-significant change in SF for dose between generations. This contrasts with UPCI-SCC-090 where the 2nd generation displayed a rapid increase in sensitivity after a 4 Gy irradiation fraction and was unable to form colonies in a 3rd generation.

### 3.3. Linear Quadratic Modelling; α/β Ratios

LQ modelling of parameters α, β and resulting ratios, exhibited highly varied values. The α and β ranges were similar irrespective of HPV status (Table 2). For confidence intervals of α and β values and R^2^ of α/β ratios please see Appendix B.

**Figure 5:**
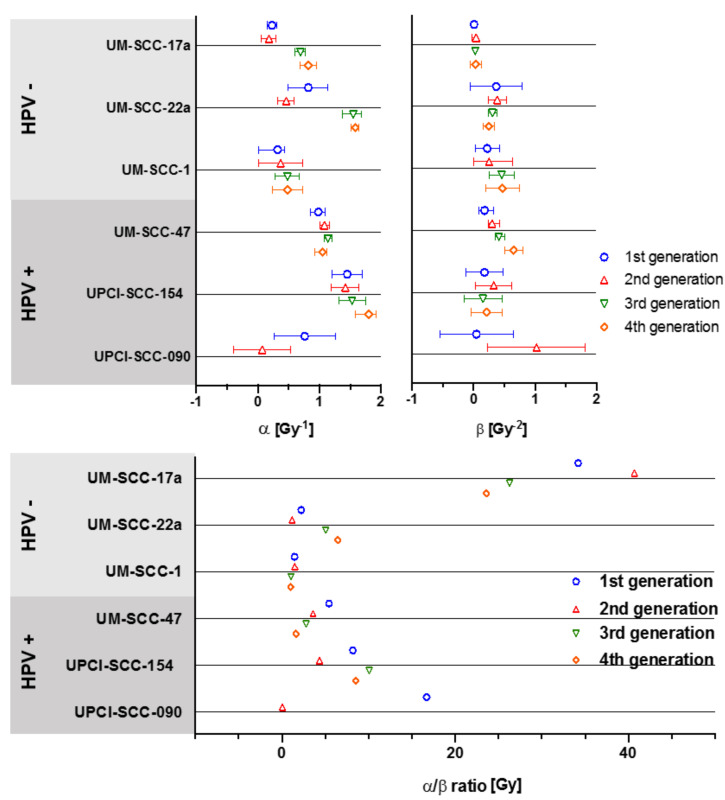
Changes in α, β and α/β ratios for 4 generations of each cell line. Error bars presented as 95% CI.

Values for α varied greatly between the 6 cell lines, ranging from 0.07 Gy^−1^ for UPCI-SCC-090 2nd generation, to 1.80 Gy^−1^ for UPCI-SCC-154 4th generation (Figure 5). Largest change in α values between generations was in the cell lines UPCI-SCC-090, UM-SCC-22a, and UM-SCC-17a. Values for β varied less by generational change and only the most sensitive line, UPCI-SCC-090, showed large change from 0.046 to 1.023 Gy^−2^ with the 2nd generation (Figure 4). The overall range for α/β ratios was as high as 40.68 Gy for the 2nd generation of UM-SCC-17a, to 0.06 Gy in the 2nd generation of UPCI-SCC-090. Cell lines UM-SCC-47, UM-SCC-1 and UM-SCC-22a displayed late responding traits with α/β less than 6 Gy. UPCI-SCC090, UPCI-SCC-154 and UM-SCC-17a showed early response characteristics and had greater inherent α/βs ranging from 8.19 to 34.19 Gy.

### 3.4. Comparison of Parameters by HPV Status

**Figure 6:**
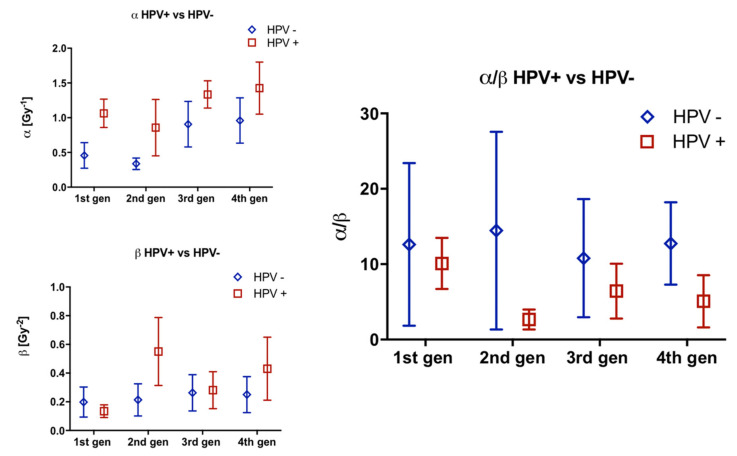
Comparison of α, β and α/β ratios by HPV group for 4 generations. Error bars represent SEM.

Comparison of α and β values and resulting α/β ratios between the HPV groups, for each generation, showed no statistical significance at any stage (Figure 6). Changes in values over the 4 generations was mixed and no clear distinction between the HPV groups was found.

## 4. Discussion

HPV positive HNSCCs show significantly better clinical outcomes and the clearly different responses to treatment raises the question of whether the 2 etiological groups should be treated the same [11,30]. The importance of describing the radiosensitivities in HNSCC lies in optimizing radiation dose for tumor control with the least normal tissue complications. Given the younger average age of patients in the better prognosis HPV positive group, these patients may live longer with a consequently higher likelihood for developing secondary effects from treatment. Many of the data that characterize the differences between cases of HNSCC according to HPV status is clinical [31]. The evident lack of biological mechanisms to explain the heightened sensitivity of HPV positive HNSCC to treatment further complicates the understanding of the differential effects of treatment according to HPV status. This study has gone beyond the investigation of inherent radiosensitivity in previously unirradiated HNSCC cell lines to develop generational cultures of cells that have survived 4 Gy irradiation fractions and redeveloped populations for subsequent re-irradiation and clonogenic assay. This has been done to simulate an approximation of the fractionated radiation therapy delivered to patients and thereby measure any changes in radiosensitivity with the progression of treatment. Additionally, we aimed to identify any distinction between the HPV groups in response to fractional irradiation and to compare changes in radio-responsiveness that may be useful in discriminating between HPV groups in terms of an approach to radiation therapy. Grouping of the 6 cell lines by HPV status, to compare SF for a given radiation dose, shows HPV positive lines to be significantly more sensitive in the previously unirradiated cells (1st generation). This is consistent with previous reports of the greater inherent radiosensitivity of HPV positive HNSCC cells where possible mechanisms include the retention of wild type p53 and the inhibition of homologous DNA repair [20,21,22,32]. Further to this, our findings show the differences in radiosensitivity between the groups remains mostly consistent with fractionation in the subsequent 3 generations. Anatomically, it was the 2 cell lines derived from laryngeal and hypopharyngeal tissue, UM-SCC-17a and UM-SCC-22a respectively that demonstrated a decrease in radiosensitivity from the 1st to the 2nd generation. The HPV positive cell lines, all derived from the tongue, and the HPV negative UM-SCC-1, derived from floor of mouth, displayed only increasing radiosensitivity with subsequent generations, albeit at different rates.

Radiosensitivity, as determined by SF as a function of absorbed dose, generally increased in all cell lines with subsequent generations. Two points of exception however, were the 2nd generations of UM-SCC-17a and UM-SCC-22a (both HPV negative cell lines) which showed increased radioresistance following 4 Gy irradiation, before the 3rd and 4th generations became more radiosensitive. Determining the intrinsic radiosensitivity of tumor cells is elemental to modelling for prediction of response and dose optimization. Cancers may display change in response to therapy during the course of treatment [33,34,35,36]. Modelling of radio-responsiveness, using SF as the measure of radiosensitivity, is based on the premise that cell killing at low doses results from a predominance of single lethal hits, producing a linear expression described by the parameter α (Gy^−1^) as a function of dose. With increasing dose, multiple hit cell killing accelerates as a quadratic function, described by the parameter β (Gy^−2^). The significance of these parameters, and in particular their ratio α/β, lies in the prediction of response to radiation and optimization of fractionation schedules for a total dose. The α/β is the most useful radiobiological parameter in radiotherapy [18]. This is illustrated by the effect tissue α/β has on the BED received in fractional doses. For example, using the equation for BED described previously, one tissue with an α/β of 10 Gy and another with 2 Gy, receiving the same fractionation schedule, 30 doses of 2 Gy, would have a BED of 72 and 120 Gy respectively. For head and neck cancers, radiotherapy commonly assumes an α/β of around 10 Gy, meaning a working assumption that these tumors are early responding and exhibit less change in radiosensitivity with alteration in fractional dose. Our findings of α/β in the 1st generation of the 6 cell lines however, show these cells to be of broadly mixed sensitivity by α/β, ranging from 1.45 Gy for UM-SCC-1 to 34.19 Gy for UM-SCC-17a which are both HPV negative. This high degree of heterogeneity for HNSCC was also reported by van Leeuwen, Oei (17) in a systematic review of published α/βs, finding a range from −83.6 Gy to 30.0 Gy. Their study quantified heterogeneity among tumor groups by the *I^2^* statistic, where an *I^2^* measure >75% is considered highly heterogeneous. The I^2^ for head and neck cancers was 87%*,* consistent with the diverse results we have observed in vitro. Both the tumor site and histology are of major influence on the cancer’s α/β and given the variable nature of these aspects in HNSCC, the observed heterogeneity may not be surprising [23]. Additionally, there was no clear trend in the changes of α/βs in subsequent generations of cell lines or distinction in α/β values between the HPV groups. The range in heterogeneity that encompasses both HPV groups shows very mixed fractionation sensitivity and calls into question the assumption of an α/β of 10 Gy for head and neck cancers. Categorizing HNSCC by a single α/β value may not be feasible given their biological diversity and the finding of mixed radiobiological values between HPV groups does not facilitate stratification along these lines. Although corroborating earlier findings of greater radiosensitivity in the HPV positive group, the significance of this study is in demonstrating a high level of heterogeneity in the radiobiology of head and neck cancers that extends across both HPV groups and challenges the assumption that the radio-responsiveness in HNSCC may be characterized by a single α/β of 10 Gy. These findings emphasize the need to be able to determine individual radiosensitivities to optimize and personalized therapy for HNSCC patients. In vitro findings of mixed chemosensitivity and radiosensitivity according to HPV status has been previously described by Nagel, Martens-de Kemp [14] looking at cell survival following single irradiation plus cisplatin and cetuximab, using fluorescent cell viability and clonogenic assay. In their study, 4 HPV positive and 14 HPV negative cell lines were investigated. Inherent in vitro treatment sensitivity displayed by these lines showed no correlation with the marked differences in clinical treatment response according to HPV status [14]. Limitations of our study include the low number of cell lines used to represent the 2 HPV groups, which is an inevitable constraint for in vitro work. Nevertheless, wide variations in radiobiological parameters both between cell lines and the sequentially irradiated generations of cells within cell lines (irrespective of HPV status) were demonstrated. Also, these measures are an intrinsic property of the tumor cells and do not take account of external factors such as hypoxia, immune cell infiltrates, and other microenvironmental influences. Although the study highlights the importance of being able to assess radio-responsiveness measured by individual clonogenic assays, these assays are not suitable for routine clinical application because of the length of time (~2 weeks) to assess radiosensitivity and because not all cells will manifest in vitro clonogenic growth.

## 5. Conclusions

Although the study found, similar to previous reports, that HPV positive cell lines as a group are more radiosensitive, we also observed broad heterogeneity in radiosensitivity irrespective of HPV group, illustrating the difficulty in stratifying patients for radiotherapy by HPV status. Specific findings from the study are:

- All cell lines displayed increasing radiosensitivity with repeated 4 Gy radiation dose fractions except the 2nd generations of UM-SCC-17a and UM-SCC-22a which showed a transient increase in radioresistance.
- Comparison of SF as a function of dose between cells of the 2 HPV groups found that HPV positive cells were significantly more radiosensitive across all generations.
- Broad heterogeneity was found for the α and β parameters from LQ modelling across all 6 cell lines without any distinct association found according to HPV status.
- The inherent α/β for each of the 6 cell lines, and the changes in this ratio with 4 Gy radiation dose fractions, did not describe any fractional sensitivity among HNSCC cell lines according to HPV status.

These observations do not support stratifying radiation therapy by HPV status. Moreover, these findings suggest that factors other than intrinsic radiosensitivity contribute more substantially to the observed differences in treatment responsiveness between HNSCC patients based on HPV status. Future work to measure radiosensitivity in progressive response to fractionation would require development of in vivo studies to assess the contribution of exogenous factors such as hypoxia, microenvironment, and immunology on response to radiation.

## Figures and Tables

**Table 1:** HNSCC cell line characteristics.

Cell Line	Anatomy	HPV Status	TP53 Mutation	Gender	Age
UM-SCC-17a	Laryngeal	Negative	Missense	F	48
UM-SCC-22a	Hypopharynx	Negative	Missense	F	
UM-SCC-1	Floor of mouth	Negative	Splice site	M	73
UPCI-SCC-090	Base of tongue	Positive	Wild type	M	46
UPCI-SCC-154	Tongue	Positive	Wild type	M	54
UM-SCC-47	Lateral tongue	Positive	Wild Type	M	53

TP 53. mutational status of each of the cell lines was determined from [24,25,26].

**Table 2:** Range of α and β values and α/β ratios by HPV status over 4 generations.

	HPV Negative	HPV Positive
**α values**	0.18–1.58 Gy^−1^	0.07–1.80 Gy^−1^
**β values**	0.007–0.470 Gy^−2^	0.046–1.023 Gy^−2^
**α/β ratios**	1.01–40.68 Gy	0.06–16.69 Gy

## Appendix A

**Table A1:** Significance of intergenerational change in SF. Change in SF between consecutive generations for dose. *p*-value by multiple *t* test. Red figures denote significance.

Gen vs. Gen/Gy	0.5 Gy	1 Gy	2 Gy	4 Gy	6 Gy
UM-SCC-17a 1st v 2nd gen	0.4782	0.1412	0.0280429	0.00001124	0.6694
UM-SCC-17a 2nd v 3rd gen	0.2387	0.01622	0.00000004	0.00000001	0.00000002
UM-SCC-17a 3rd v 4th gen	0.8392	0.8392	0.4003	0.020931	0.02073604
UM-SCC-22a 1st v 2nd gen	0.7873	0.017954	0.3044	0.7873	0.3748
UM-SCC-22a 2nd v 3rd gen	0.1184	0.000013	0.1184	0.2689	0.2689
UM-SCC-22a 3rd v 4th gen	0.8940	0.5588	0.0013		
UM-SCC-1 1st v 2nd gen	0.9432	0.0845	0.3980	0.9432	
UM-SCC-1 2nd v 3rd gen	0.4782	0.0583	0.0266	0.0179	
UM-SCC-1 3rd v 4th gen	0.9688	0.9688	0.9688		
UM-SCC-47 1st v 2nd gen	0.6547	0.2562	0.1635	0.0030	
UM-SCC-47 2nd v 3rd gen	0.8018	0.8018	0.0897		
UM-SCC-47 3rd v 4th gen	0.9749	0.4743	0.7982		
UPCI-SCC-154 1st v 2nd gen	0.9744	0.9744	0.5995	0.9744	0.0001
UPCI-SCC-154 2nd v 3rd gen	0.9973	0.9973	0.9973	0.7699	0.8380
UPCI-SCC-154 3rd v 4th gen	0.6816	0.6929	0.6246	0.0000010	0.0634
UPCI-SCC-090 1st v 2nd gen	0.9565	0.0004	0.0000009	0.0000197	0.0000009
UPCI-SCC-090 2nd v 3rd gen					
UPCI-SCC-090 3rd v 4th gen					

## Appendix B

**Table A2:** Modelling parameters and α/β by R^2^.

Cell Line and Generation	α	95% Confidence	β	95% Confidence	R^2^	α/β
SCC-17a 1st	0.23	(0.1542, 0.3131)	0.007	(−0.012, 0.026)	0.956	34.19
SCC-17a 2nd	0.18	(0.053, 0.298)	0.004	(−0.024, 0.033)	0.969	40.68
SCC-17a 3rd	0.69	(0.6048, 0.7655)	0.026	(−0.0222, 0.07431)	0.996	26.30
SCC-17a 4th	0.82	(0.6869, 0.9523)	0.035	(−0.061, 0.1304)	0.999	23.61
SCC22A-1st	0.82	(0.4963, 1.142)	0.368	(−0.05686, 0.7924)	0.962	2.23
SCC22A-2nd	0.46	(0.3241, 0.5897)	0.387	(0.2351, 0.5396)	0.995	1.18
SCC22A-3rd	1.55	(1.372, 1.688)	0.306	(0.237, 0.3755)	1.000	5.06
SCC22A-4th	1.58	(1.513,1.644)	0.245	(0.1502, 0.3396)	1.000	6.44
SCC1-1st	0.32	(0.0093, 0.428)	0.220	(0.0319, 0.4226)	0.977	1.45
SCC1-2nd	0.37	(0.0151, 0.729)	0.250	(0.0012, 0.6339)	0.995	1.49
SCC1-3rd	0.48	(0.2855, 0.6666)	0.456	(0.2487, 0.6633)	0.996	1.04
SCC1-4th	0.48	(0.2314, 0.7225)	0.470	(0.197, 0.7439)	0.986	1.01
SCC154-1st	1.45	(1.205, 1.693)	0.177	(−0.1226, 0.4765)	0.992	8.19
SCC154-2nd	1.42	(1.194, 1.643)	0.328	(0.03136, 0.6241)	0.995	4.33
SCC154-3rd	1.53	(1.313, 1.754)	0.152	(−0.1562, 0.4609)	0.996	10.07
SCC154-4th	1.80	(1.582,1.913)	0.211	(−0.0408, 0.4624)	0.998	8.54
SCC090-1st	0.76	(0.2703, 1.254)	0.046	(−0.5512, 0.6425)	0.851	16.69
SCC090-2nd	0.07	(−0.3983, 0.5297)	1.023	(0.2293, 1.816)	0.992	0.06
SCC47-1st	0.98	(0.859, 1.091)	0.180	(0.0919,0.3315)	0.998	5.42
SCC47-2nd	1.08	(1,1.156)	0.300	(0.2388,0.4188)	0.999	3.59
SCC47-3rd	1.14	(1.075,1.201)	0.410	(0.3481,0.5017)	1.000	2.78
SCC47-4th	1.05	(0.924,1.116)	0.650	(0.5143,0.8034)	0.999	1.62

## References

[1] Leemans C.R., Braakhuis B.J.M., Brakenhoff R.H. (2011). Response to correspondence on the molecular biology of head and neck cancer. Nat. Rev. Cancer.

[2] Syrjänen S. (2005). Human papillomavirus (HPV) in head and neck cancer. J. Clin. Virol..

[3] Pan C., Issaeva N., Yarbrough W.G. (2018). HPV-driven oropharyngeal cancer: Current knowledge of molecular biology and mechanisms of carcinogenesis. Cancers Head Neck.

[4] Chaturvedi A.K., Anderson W.F., Lortet-Tieulent J., Curado M.P., Ferlay J., Franceschi S., Rosenberg P.S., Bray F., Gillison M.L. (2013). Worldwide Trends in Incidence Rates for Oral Cavity and Oropharyngeal Cancers. J. Clin. Oncol..

[5] Berger M.H., Haidar Y.M., Bitner B., Trent M., Tjoa T. (2019). Practice patterns and knowledge among California pediatricians regarding human papillomavirus and its relation to head and neck cancer. Am. J. Otolaryngol..

[6] A Ratko T., Douglas G., De Souza J.A., E Belinson S., Aronson N. (2014). Radiotherapy Treatments for Head and Neck Cancer Update.

[7] Lassen P., Eriksen J.G., Krogdahl A., Therkildsen M.H., Ulhøi B.P., Overgaard M., Specht L., Andersen E., Johansen J., Andersen L.J. (2011). The influence of HPV-associated p16-expression on accelerated fractionated radiotherapy in head and neck cancer: Evaluation of the randomised DAHANCA 6&7 trial. Radiother. Oncol..

[8] Fakhry C., Zhang Q., Nguyen-Tan P.F., Rosenthal D.I., El-Naggar A., Garden A.S., Soulieres D., Trotti A., Avizonis V., Ridge J.A. (2014). Human Papillomavirus and Overall Survival After Progression of Oropharyngeal Squamous Cell Carcinoma. J. Clin. Oncol..

[9] Rosenthal D.I., Harari P.M., Giralt J., Bell D., Raben D., Liu J., Schulten J., Ang K.K., Bonner J.A. (2016). Association of Human Papillomavirus and p16 Status with Outcomes in the IMCL-9815 Phase III Registration Trial for Patients with Locoregionally Advanced Oropharyngeal Squamous Cell Carcinoma of the Head and Neck Treated With Radiotherapy With or Without Cetuximab. J. Clin. Oncol..

[10] Gillison M.L., Chaturvedi A.K., Anderson W.F., Fakhry C. (2015). Epidemiology of Human Papillomavirus–Positive Head and Neck Squamous Cell Carcinoma. J. Clin. Oncol..

[11] Masterson L., Moualed D., Liu Z.-W., Howard J.E., Dwivedi R.C., Tysome J.R., Benson R., Sterling J.C., Sudhoff H., Jani P. (2014). De-escalation treatment protocols for human papillomavirus-associated oropharyngeal squamous cell carcinoma: A systematic review and meta-analysis of current clinical trials. Eur. J. Cancer.

[12] Ihloff A., Petersen C., Hoffmann M., Knecht R., Tribius S. (2010). Human papilloma virus in locally advanced stage III/IV squamous cell cancer of the oropharynx and impact on choice of therapy. Oral Oncol..

[13] Maggiorella L., Barouch G., Devaux C., Pottier A., Deutsch É., Bourhis J., Borghi E., Lévy L. (2012). Nanoscale radiotherapy with hafnium oxide nanoparticles. Futur. Oncol..

[14] Nagel R., Kemp S.M.-D., Buijze M., Jacobs G., Braakhuis B.J., Brakenhoff R.H. (2013). Treatment response of HPV-positive and HPV-negative head and neck squamous cell carcinoma cell lines. Oral Oncol..

[15] Joiner M.C., van Der Kogel A., Joiner M.C., van Der Kogel A.J. (2018). Basic Clinical Radiobiology.

[16] Hall E.J., Giaccia A.J. (2006). Radiobiology for the Radiologist.

[17] Van Leeuwen C.M., Oei A.L., Crezee J., Bel A., Franken N., Stalpers L.J.A., Kok H.P. (2018). The alfa and beta of tumours: A review of parameters of the linear-quadratic model, derived from clinical radiotherapy studies. Radiat. Oncol..

[18] Murray D., McBride W.H., Schwartz J.L. (2014). Radiation Biology in the Context of Changing Patterns of Radiotherapy. Radiat. Res..

[19] Fowler J.F. (1989). The linear-quadratic formula and progress in fractionated radiotherapy. Br. J. Radiol..

[20] Arenz A., Ziemann F., Mayer C., Wittig A., Dreffke K., Preising S., Wagner S., Klussmann J.-P., Engenhart-Cabillic R., Wittekindt C. (2014). Increased radiosensitivity of HPV-positive head and neck cancer cell lines due to cell cycle dysregulation and induction of apoptosis. Strahlenther. Onkol..

[21] Dok R., Kalev P., Van Limbergen E.J., Asbagh L.A., Hauben E., Sablina A.A., Nuyts S., Vazquez I. (2014). p16INK4a Impairs Homologous Recombination-Mediated DNA Repair in Human Papillomavirus-Positive Head and Neck Tumors. Cancer Res..

[22] Rieckmann T., Tribius S., Grob T., Meyer F., Busch C.-J., Petersen C., Dikomey E., Kriegs M. (2013). HNSCC cell lines positive for HPV and p16 possess higher cellular radiosensitivity due to an impaired DSB repair capacity. Radiother. Oncol..

[23] Geh J.I., Bond S., Bentzen S.M., Glynne-Jones R. (2006). Systematic overview of preoperative (neoadjuvant) chemoradiotherapy trials in oesophageal cancer: Evidence of a radiation and chemotherapy dose response. Radiother. Oncol..

[24] Sano D., Xie T.-X., Ow T.J., Zhao M., Pickering C.R., Zhou G., Sandulache V.C., Wheeler D.A., Gibbs R.A., Caulin C. (2011). Disruptive TP53 mutation is associated with aggressive disease characteristics in an orthotopic murine model of oral tongue cancer. Clin. Cancer Res..

[25] Brenner J.C., Graham M.P., Kumar B., Bs L.M.S., Kupfer R., Lyons R.H., Bradford C.R., Carey T.E., Bs M.P.G. (2009). Genotyping of 73 UM-SCC head and neck squamous cell carcinoma cell lines. Head Neck.

[26] White J.S., Weissfeld J.L., Ragin C.C.R., Rossie K.M., Martin C.L., Shuster M., Ishwad C.S., Law J.C., Myers E.N., Johnson J.T. (2007). The influence of clinical and demographic risk factors on the establishment of head and neck squamous cell carcinoma cell lines. Oral Oncol..

[27] Moore C.S., Wood T.J., Cawthorne C., Hilton K.L., Maher S., Saunderson J.R., Archibald S.J., Beavis A.W. (2016). A method to calibrate the RS 2000 X-ray biological irradiator for radiobiological flank irradiation of mice. Biomed. Phys. Eng. Express.

[28] Ma C.-M.C., Coffey C.W., DeWerd L.A., Liu C., Nath R., Seltzer S.M., Seuntjens J. (2001). AAPM protocol for 40-300 kV X-ray beam dosimetry in radiotherapy and radiobiology. Med. Phys..

[29] Schneider C.A., Rasband W.S., Eliceiri K.W. (2012). NIH Image to ImageJ: 25 years of image analysis. Nat. Methods.

[30] Lassen P. (2010). The role of Human papillomavirus in head and neck cancer and the impact on radiotherapy outcome. Radiother. Oncol..

[31] Lewis A., Kang R.S., Levine A., Maghami E. (2015). The New Face of Head and Neck Cancer: The HPV Epidemic. Oncology.

[32] Kimple R.J., Smith M.A., Blitzer G.C., Torres A.D., Martin J.A., Yang R.Z., Peet C.R., Lorenz L.D., Nickel K.P., Klingelhutz A.J. (2013). Enhanced Radiation Sensitivity in HPV-Positive Head and Neck Cancer. Cancer Res..

[33] Enriquez-Navas P.M., Wojtkowiak J.W., Gatenby R.A. (2015). Application of Evolutionary Principles to Cancer Therapy. Cancer Res..

[34] Gatenby R.A., Silva A.S., Gillies R.J., Frieden B.R. (2009). Adaptive therapy. Cancer Res..

[35] Baskar R., Dai J., Wenlong N., Yeo R., Yeoh K.-W. (2014). Biological response of cancer cells to radiation treatment. Front. Mol. Biosci..

[36] Wang J.-S., Wang H.-J., Qian H. (2018). Biological effects of radiation on cancer cells. Mil. Med. Res..

